# Incidental catch of loggerhead sea turtles (*Caretta caretta*) along the Sicilian coasts by longline fishery

**DOI:** 10.7717/peerj.5392

**Published:** 2018-08-07

**Authors:** Santo Caracappa, Maria Flaminia Persichetti, Antonio Piazza, Giulia Caracappa, Antonino Gentile, Sandra Marineo, Daniela Crucitti, Marco Arculeo

**Affiliations:** 1 Istituto Zooprofilattico Sperimentale della Sicilia, Palermo, Italy; 2 Dipartimento di Scienze e Tecnologie Biologiche, Chimiche e Farmaceutiche, University of Palermo, Palermo, Italy

**Keywords:** *Caretta caretta*, Loggerhead turtle, Hooks, Strandings, Incidental take, Sicily, Longline fishing, Mediterranean Sea

## Abstract

It has been estimated that 44,000 *Caretta caretta* turtles die every year due to anthropomorphic activity in the Mediterranean Sea, and that longline fishing is one of the most significant causes of mortality. A total of 482 specimens of *C. caretta* were rescued from different parts of the Sicilian coast (Mediterranean Sea) from 2014 to 2016. The most numerous stranding was recorded during the spring and summer seasons, mainly along the north and eastern coasts of Sicily. The curved carapace length for all the specimens ranged from between 19 and 95 cm and most of these were young or sub adults. The highest number of strandings was recorded in 2014 and 2015, with 206 and 169 individuals, respectively. A total of 66 live specimens out of 239 were successfully rehabilitated and released after surgery or drug therapy; fishing hooks were found in 129 specimens in different parts of the digestive tract with greater frequency in the oesophagus (47.3%) followed by the gut (24.8%), stomach (14.7%), and mouth (13.2%). This paper will highlight the incidence of the incidental catch by longline fishing of *C. caretta* along the Sicilian coasts and also relate the size of ingested hooks to the size of examined specimens.

## Introduction

One of the primary causes of loggerhead sea turtle stranding is incidental capture by fishing gear with particular reference to longline fishing (Camiñas & De La Serna, 1995; Laurent et al., 2001; Lewison, Freeman & Crowder, 2004; Camiñas-Hernández et al., 2006; Casale, Freggi & Rocco, 2008; Di Bello et al., 2013; Blasi & Mattei, 2017). Other threats in the Mediterranean Sea include the destruction of reproductive habitats (Casale & Margaritoulis, 2010), collisions with boats, and intentional killing (Tomás et al., 2008; Casale et al., 2010; Casale, 2011). Small to large-scale fishing fleets, mainly using longline, are primarily responsible for the bycatch of loggerhead sea turtles in the Mediterranean Sea and in other parts of the world; other dangers originate from set nets and bottom trawling (Santos et al., 2013; Casale, 2011; Wallace et al., 2010). The different impact of fishing gear also depends on the structures and habits of fishing ports, and the areas where turtles and fishing gear are obliged to cohabit. Pelagic longline fishing gear which targets dolphinfish, tuna, amberjack, and swordfish is also problematic for sea turtles (Stokes, Epperly & McCarthy, 2012). The bycatch threat from these types of fishing gear poses a serious problem for the survival and conservation of marine turtles, as well as for larger fish and marine mammals with long life-cycles, requiring years to reach sexual maturity.

An estimated 52,340 capture occurred in the Mediterranean in 2014 due to incidental capture by fishing gear (Lucchetti, Vasapollo & Virgili, 2017) and approximately 44,000 loggerhead sea turtles died each year (Casale, 2011). This number can also be said to be underestimated if the unmonitored trawlers and small fishing vessels, operating off the African coasts in the Mediterranean, are also taken into consideration. Moreover, many European anglers do not often report incidental catches and, during the Mediterranean summer season, there is intense recreational fishing activity with an elevated use of longlines. It is, therefore, not easy to precisely estimate the number of people fishing recreationally throughout the year: whilst this activity is regulated by Italian law, enforcement is inadequate. It has been reported the number of those engaging in recreational fishing may be close to one million (i.e., http://www.biologiamarina.eu/IncPescaSportiva/Inchiesta%20pesca%20sportiva.html; http://www.datiopen.it/it/opendata/Numero_dei_pescatori_residenti_di_pesca_sportiva_per_regione_al_2014).

According to the last IUCN assessment, the Mediterranean subpopulation of loggerhead sea turtles is listed as being of “Least Concern” (http://www.iucnredlist.org/details/83644804/0). And, despite the fact that the loggerhead turtle has been widely studied within and beyond the Mediterranean, there is a paucity of studies addressing a reduction of the impact of fishing on this species (see http://www.tartalife.eu/it; http://cordis.europa.eu/project/rcn/39467_en.html). To date, several projects, supported by regional/national administrations or various European community funds, have been available to agree upon solutions which would reduce the incidental catch of sea turtles. And to date, there have been few initiatives by the Italian government, many of which have proved ineffective.

Considering that the loggerhead sea turtle is the most abundant sea turtle in the Mediterranean Sea, data regarding its interactions with fishing gear are fundamental for evaluating the effects of these interactions in different areas. The objective of this paper is to make available new information regarding the incidental bycatch of loggerhead sea turtles along the Sicilian coast, and to further our understanding of the impact of this phenomenon in the Mediterranean. Various considerations regarding the incidence of the hooks in different parts of the digestive system and the relationship between ingested hook size and turtle size will also be discussed.

## Methods

### Study area

**Figure 1:**
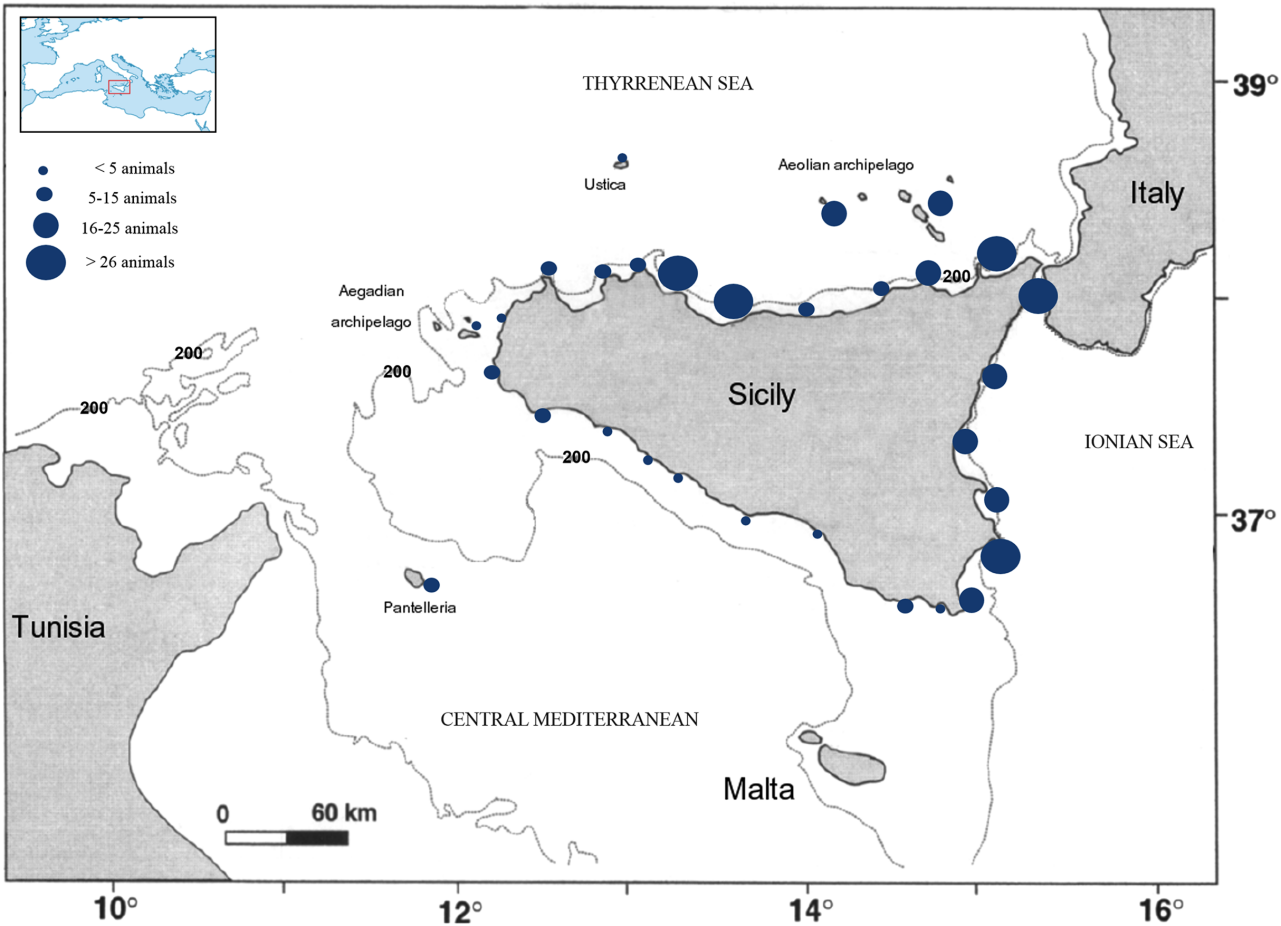
Origin of the specimens of *C. caretta* stranded. The size of circles represents the number of strandings found along the Sicilian coasts.

Sicily is the largest island (area of 25,711 km^2^) in the Mediterranean Sea, being surrounded to the north by the Tyrrhenian Sea, the south by the Strait of Sicily, and to the east by the Ionian Sea (Fig. 1). The diverse physiognomy of the three Sicilian coasts is reflected in the different distribution of fishing gear with the greatest presence, for example, in terms of absolute quantity, being located on the northern coast (Pernice, 2013). Moreover, the Sicilian region comprises many other small islands, and some of these form small archipelagos (e.g., the Pelagie, Aeolian, and Egadi Islands).

Sicily is represented by a different number of fishing vessels and according to the “Community Fishing Fleet Register” of the European Union in December 2013 (https://data.europa.eu/euodp/data/dataset/the-community-fishing-fleet-register), there are 48 fishing ports in Sicily with a total number of 2,892 boats (Pernice, 2013). The type of fishing gear most commonly used by the Sicilian fishing fleet are longline (43%), purse seine (27%), and trawler (20%) (Pernice, 2013).

### Sampling collection

From 2014 to 2016, 482 specimens of loggerhead sea turtle were rescued from different parts of the Sicilian coast (Fig. 1). This ran concurrent with a monitoring program relating to turtle strandings along the Sicilian coasts, which was started in 2014 by the Istituto Zooprofilattico Sperimentale della Sicilia (IZS). In the majority of cases, the specimens, had been sighted and collected by the Coast Guard as part of routine checks during the year, which were performed along the coasts of Sicily. Usually the Coast Guard carries out the monitoring activities of the coasts periodically, at least once a week during the winter period, and almost daily for the rest of the year. In some cases when individuals were observed to be in difficulty, stranded or they were found by fishermen or bathers, the latter invariably alerted the Coast Guard. Subsequently, the Coast Guard contacted the relevant personnel of the Regional Center of the Recovery for Sea Turtles at the Veterinary Public Health Institute of Sicily (IZS Sicily), located in Palermo; they are engaged in the recovery and transportation of loggerhead turtles to the Center. The IZS Sicily is the only Centre authorized by the Region of Sicily and the Ministry of Health (regional low n. 6067/2013 and national low n. 96 del April 26, 2016) to perform surgery and rehabilitation treatments. Where possible, the body weight, curved carapace length (CCL notch to tip), width, and the plastron (Straight Plastron Length and Straight Plastron width) were recorded, according to Wyneken (2001). Turtle sex was determined by different means: (i) a visual examination of the gonads during necropsy, (ii) tail length, used only for individuals with CCL >50 cm, and (iii) laparoscopy according to the guidelines described by Wyneken (2001), Wyneken et al. (2007), and Wood et al. (1983). Immediately after their recovery, health checks were provided and treatment (if necessary) provided on live animals who required further treatment. Those in need of specialized care were hospitalized until they could be released. In all live individuals, radiographic investigations were performed to identify the presence of metallic objects, such as fishing hooks in the mouth, oesophagus, or elsewhere in the digestive tract. When required, objects were surgically removed while the animal was under general anesthesia. Whenever possible, turtles found floating dead in the water or beached, were transferred to IZS Sicily for necropsy to determine the cause of death. If fishing hooks were found, the length and position of each hook in different parts of the digestive system was recorded. Hook size (length in millimeter) ranged from 20 to 80 mm. The different sizes of hooks were grouped into three different class sizes (21–40, 41–60, and 61–80 mm) and related to the dimensions of individual turtles. It was very difficult to convert hooks dimensions in millimeter into the commercial scales reported by different brands of hooks. Often no correspondence was observed and it was, therefore, decided to report hook dimensions in length (millimeter).

## Results

**Figure 2:**
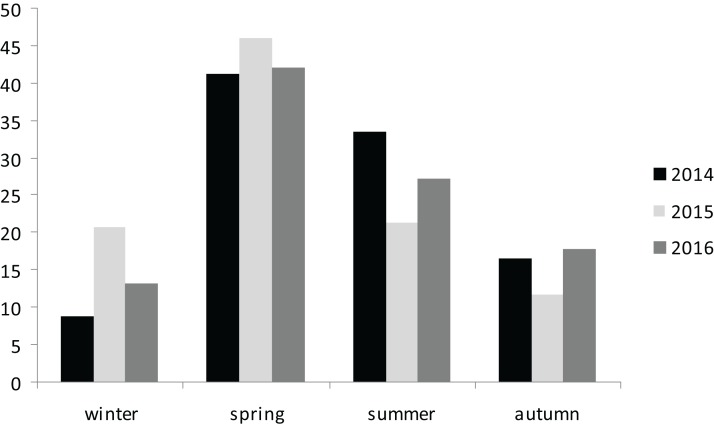
Seasonal percentage *C. caretta* stranded along Sicilian coasts.

Extrapolating from the 3-year dataset in this study demonstrated that the greatest number of strandings occurred along the north and east coasts of Sicily (Fig. 1), with spring and summer being peak seasons for strandings (Fig. 2; Table 1). Of the 482 individuals, 239 were recovered alive, while 243 were found dead, floating on the water or stranded (117 of these individuals were severely decomposed). The CCL of all (*n* = 482) observed loggerhead sea turtles ranged from between 19 and 95 cm (Table 1). During the three years survey period, the highest number of strandings was recorded in the years 2014 and 2015, with 206 and 169 individuals respectively. The strandings included animals of all size classes from juvenile to adult. If the dimensions of all individuals was considered, the sample contained predominantly young specimens, compared to adults (CCL >70 cm) (Table 1). A total of 69 (62%) females and 42 (38%) males were recorded; the sex of the other specimens could not be determined due to: (i) advanced stage of decomposition of the internal organs; (ii) impossibility of necropsy; or (iii) the young age of the specimens. The mean CCL of male turtles was 52.4 cm (SD ± 11.2) of CCL, and for females it was 52.6 cm (SD ± 9.7). Of the 243 dead turtles, the causes of death were varied, ranging from the ingestion of plastic refuse, fishing line and hooks and boat strikes; it was difficult to determine the cause of death on an individual basis. Indeed, the presence of ingested plastic in many cases, together with the presence of hooks or line, made it difficult to distinguish which factor had caused death. Moreover, the stage of decomposition also obfuscated the exact cause of death.

**Table 1:** Number of individuals stranded alive, dead and released after undergoing treatment during the 2014–2016 period. The minimum and maximum dimensions of individuals are also reported.

	Winter	Spring	Summer	Autumn	Size class (cm)	CCL >70 (cm)
**2014**						
Live	5	24		18	19–82 (45 ± 13.5)	3
Dead	13	61	31	16	22–95 (56.2 ± 11.4)	11
Released	7	1	7	10	24–64 (42.5 ± 13.1)	
**2015**						
Live	15	27	22	11	20–78 (45.7 ± 12.6)	1
Dead	20	51	14	9	24–95 (56.2 ± 16.3)	9
Released	3	7	3	2	24–78 (44.2 ± 15.5)	
**2016**						
Live	9	29	23	15	19–71 (43.3 ± 14.1)	1
Dead	5	16	6	4	27–95 (61.1 ± 19.7)	2
Released	1	4	12	9	21–71 (49.9 ± 13.2)	

**Figure 3:**
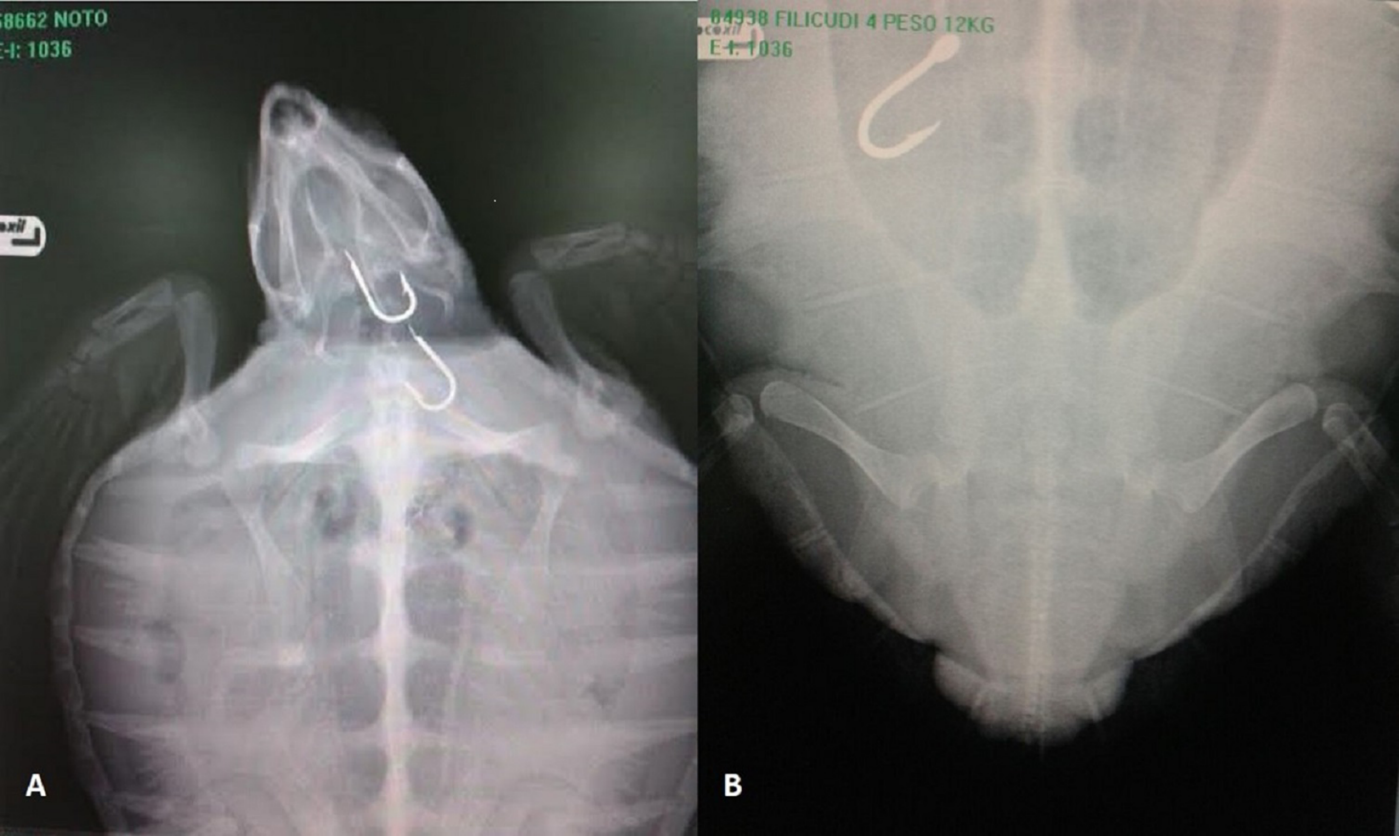
X-ray of *C. caretta*. (A) Turtle rescued in Noto locality (South of Sicily) with two hooks in the esophagus. (B) Turtle rescued in Filicudi locality (Northeast of Sicily) with one hook in the intestine.

**Figure 4:**
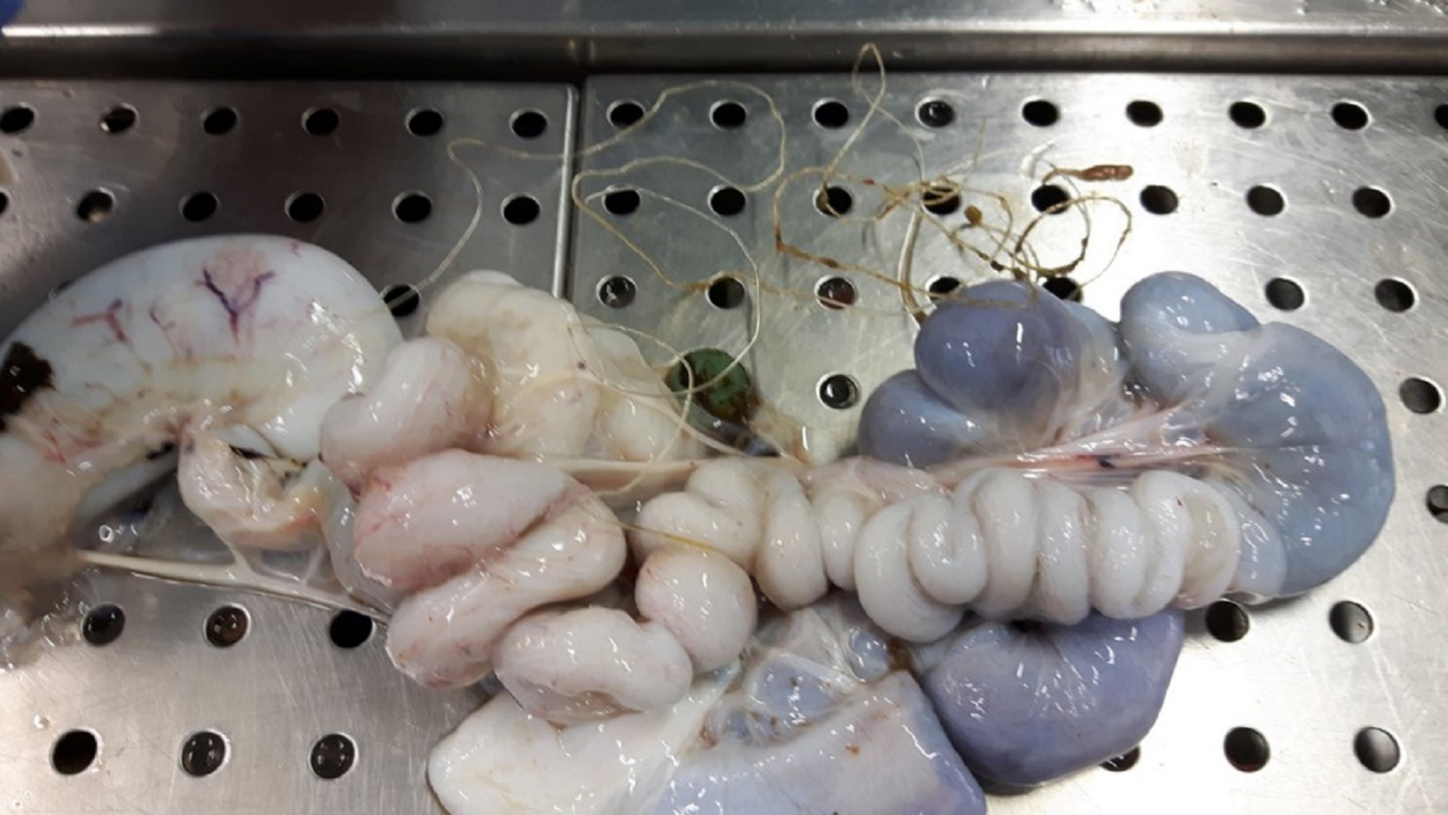
Intestinal torsion and obstruction caused by ingested line.

Of the 239 alive individuals recovered and processed at the IZS, 66 (27,6%) specimens were successfully rehabilitated and released after surgery or drug therapy (Table 1). A total of 129 specimens (105 alive and 24 dead) were found to have ingested hooks, while the remaining specimens (*n* = 110) displayed: signs of weakness, ongoing disease pathologies (25.5%), external injuries caused by accidental contact with boats (16.3%), plastic refuse (26.4%) or lengths of fishing line (31.8%) in the digestive system. Fishing hooks were observed in different parts of the digestive tract, including the oesophagus (47.3%), intestinal tract (24.8%), stomach (14.7%), and mouth (13.2%) (Fig. 3). Specifically, of the live 105 individuals who had ingested hooks, 23 specimens with hooks in their mouths or the initial part of the oesophagus underwent successful surgery and they were subsequently rehabilitated and released. However, some specimens presented with oesophageal lesions or severe perforation of the stomach or intestine, as evidenced during surgery to remove the fishing hooks, and this negatively influenced their prognosis. It has been well documented that the oesophageal wall is much thicker than that of the stomach, and that the presence of hooks in the oesophagus can manifest infections which subsequently cause systemic septicemia (Oros et al., 2005). Hooks in the stomach, can have a devastating effect on the life of loggerhead causing punctures and purulent coelomitis: this has been documented by Oros, Calabuig & Deniz (2004). Worst still, hooks were often associated with lines, which frequently caused intestinal torsion (Fig. 4), obstruction, and/or perforation of the intestinal wall with consequent severe coelomitis.

**Figure 5:**
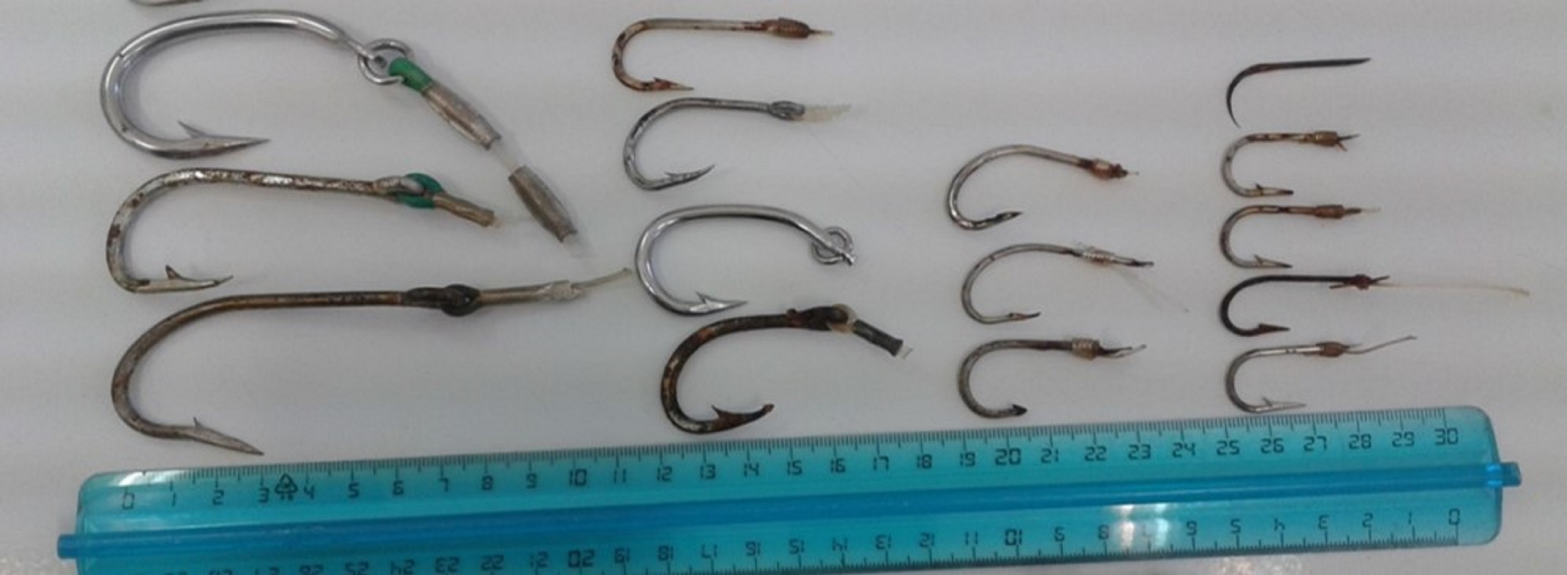
Different size of J-hooks found in different tract of the digestive system. On the left (first and second group hook sizes about 80 and 40–50 mm). On the right (first and second group hook sizes about 20 and 30 mm).

**Figure 6:**
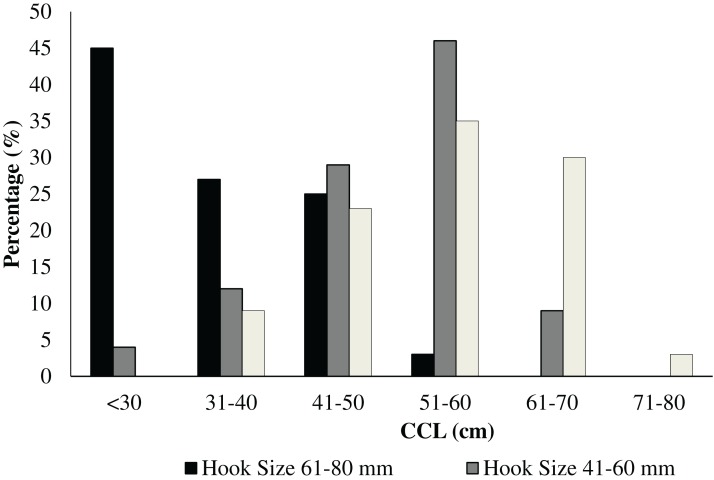
Distribution of the number and the size (range: 21–40, 41–60, and 61–80 mm) of hooks in relation to the carapacial size (CCL) in cm of the rescued *C. caretta* specimens.

Making reference to hook (Figs. 5 and 6) and specimen size (CCL), it was evident that hook dimensions were variously and according to the size of the loggerhead sea turtles (Fig. 6).

Finally, the data in this study confirmed the greatest number of stranding occurred in the spring, followed by the summer (see Fig. 2) in the 3-year period under investigation.

## Discussion

The data obtained in this study highlight the significance of incidental catch of loggerhead sea turtle by longline fishery along the coasts of Sicily. It also revealed that loggerheads frequently had hooks in the oesophagus or intestine, causing severe injuries with a high mortality rate. Observations indicated that the presence of a hook in the mouth or the initial part of the oesophagus did not usually create particular problems unless the position and size of the hook compromised feeding. The reality of having rehabilitated and released only 27.6% of individuals, highlighted the discrete results obtained with surgery or pharmacological treatments, especially when injuries from hooks or other causes are advanced. Unfortunately, this result could not be compared with others studies in the Mediterranean Sea because this research is to be a first of its kind in the field, in which the survival of stranding individuals after surgery and pharmacology has been highlighted.

Hook size and type can have a significant effect on their potential ingestion by loggerhead sea turtle, which in turn depend on the size of the individual (Stokes, Epperly & McCarthy, 2012). Moreover, the size and shape of hooks may be indicators of the foraging habits of loggerhead sea turtle as suggested by Lucchetti & Sala (2009), and also the target species which is sought. Bottom longlines with smaller hooks are usually used in shallower waters to catch species such as sparids, serranids, etc., unlike pelagic longlines with larger hooks which are used to catch swordfish, dolphinfish, amberjack, or other pelagic fishes. The data showed an evident relationship between hook size and turtle size, which suggests a strong relationship between the areas frequented by fishermen and loggerhead sea turtle. Unfortunately, given the random origin of our records and the generality of the information obtained, it is very difficult to state whether or not the area where the catch or standing occurred is a manifestation of the area where the fishing occurred. However, it is clear that the greatest number of strandings occurred along the northern and eastern coasts of Sicily where longline fishing boats predominate (Pernice, 2013). There is also a higher degree of anthropization of the coasts with a consequent impact on the marine ecosystem. Furthermore, a knowledge of size classes of loggerhead sea turtles, which are typically encountered in the neritic and pelagic phases is critical to improved our understanding of this possible relationship (hook size and of loggerhead sea turtle dimensions). Of note is that fact that the data demonstrated the majority of incidental catches were young or sub-adult individuals, thereby highlighting the need to adopt urgent measures to reduce these catches.

According to observations made by Blasi & Mattei (2017) in the Aeolian Arcipelago (southern Tyrrhenian Sea) and Lucchetti, Vasapollo & Virgili (2017) in other areas of the Mediterranean Sea, the largest number of turtles caught or stranded occurred mainly in the summer season. This probably coincided with the increase in professional fishing activities, in turn due to the increased availability of pelagic fishes. Our data partially agree with the observations of Blasi & Mattei (2017) and Lucchetti, Vasapollo & Virgili (2017) in that, in the former, a higher peak in the spring followed by the summer was observed (Fig. 2). Spring and summer are crucial seasons for this species because they coincide or are close to their breeding period. This fact must be considered in formulated mitigation measures to reduce accidental catch and consequent mortality.

When compared to other types of fishing, longline fishing in Sicily, has potentially the highest turtle bycatch rates observed for commercial fisheries. In fact, according to the “Community Fishing Fleet Register” of the European Union for 2013 and Pernice (2013) have confirmed the high potential impact which longlines may have on this species. To this must be added the considerable number of (i) recreational fishing activities, difficult to quantify; (ii) fishing vessels operating in African countries bordering the Mediterranean, that is, sources of unreported and unregulated fishing; and (iii) the potentially high post-release mortality, which is associated with long line-turtle interactions.

Recent studies have urged drastic measures for reducing bycatch and resulting mortality of this species with the deployment of a few simple guidelines (Lucchetti & Sala, 2009; Piovano, Swimmer & Giacoma, 2009; Sales et al., 2010; Stokes, Epperly & McCarthy, 2012; Sherker, 2017). The first consists of the modifications of hook type (a circle hook instead of a J-hook) and bait species. According to Gilman et al. (2006) and Watson et al. (2005), the replacement of the J-hook with a squid bait to a circle hook with other bait species (such as the mackerel) showed a significant reduction in 90% of loggerhead sea turtle bycatch. The second measure, of marginal impact, concerns the use of Turtle excluder devices in trawler nets, and the use of light bulbs in the trammel net and gillnets. This latter measure, recently adopted experimentally by Ortiz et al. (2016) in Sechura Bay, Peru and in the Mediterranean Sea (Northern Adriatic Sea) (Virgili, Vasapollo & Lucchetti, 2018), with its fishing gear, illuminated with light-emitting diodes placed every 5–10 m along the gillnet floatline, seems to be very effective in significantly reducing bycatch.

According to the Action Plan for the Conservation of Mediterranean Marine Turtles (UNEP MAP RAC/SPA, 2007), the impact of fishing activities is one of the most important anthropogenic mortality factors for loggerhead sea turtles in the Mediterranean Sea (Gerosa & Casale, 1998). These data are almost 20-years-old and, to date, there has only been one update relating to the Italian waters (Lucchetti, Vasapollo & Virgili, 2017). Greater coordination among the existing rescue centers and also greater involvement with the fishermen can contribute to reducing the capture and consequent mortality of this important species. Specifically, this can be achieved by: (i) staffing recovery and rescue centers with qualified personnel who can perform surgical procedures to safely remove hooks and (ii) reducing instances where fisherman either release animals caught with ingested hooks back into the sea or remove hooks directly with inappropriate and often harmful procedures. These precautions can contribute to reducing the bycatch mortality of this species. Furthermore, incidental capture can be reduced by improving management of the distribution of the fishing effort spatially and temporally, thereby avoiding particular areas or modifying the characteristics of longlines (i.e., circle hooks, reducing soak time, changing bait species, etc.). In addition, satellite tracking and aerial surveys could provide high resolution data, which is required to improve the coordination of fisheries management and marine spatial planning.

## Conclusion

Data relating to strandings along the Sicilian coasts provides key information regarding the seasonal distributions of loggerhead sea turtle and the area where the stranding is most like to occur. The north and east coasts of Sicily are currently the most subject to strandings, probably due to a greater human impact as well as fishing activities. Our findings on the impacts of fisheries in this region signal a need to develop mitigation measures, which are aimed at reducing the mortality of loggerhead sea turtle, and contributing data which is relevant in formulating conservation plans. Fisheries and marine protection policies should share a common goal: to achieve sustainable development through the maintenance of a flourishing economy which has respect for biological diversity. Far too often, fishing policy mainly regards aspects of resource withdrawal and, to a lesser extent, the impact of fishing on the marine environment; at its peril, current fishing policy ignores its impacts on the endangered species of cetaceans, sea turtle, sharks, to name but a few.

## Supplemental Information

10.7717/peerj.5392/supp-1 Supplemental Information 1 Figure 5 raw data.

10.7717/peerj.5392/supp-2 Supplemental Information 2 Figure 6 raw data.

## Additional Information and Declarations

### Data Availability

The following information was supplied regarding data availability: The raw data are provided in the Supplemental Files.
